# Obesity, abdominal obesity, metabolic obesity phenotypes, and *Helicobacter pylori* infection: results from NHANES 1999–2000

**DOI:** 10.1186/s12879-024-09409-7

**Published:** 2024-07-06

**Authors:** Danni Chen, Shiling Wang, Wei Yang, Hong Lu, Qian Ren

**Affiliations:** 1 The First School of Clinical Medicine, Lanzhou University, Lanzhou, 730000 China; 2 Department of Gastroenterology, the First Hospital of Lanzhou University, Lanzhou, 730000 China; 3 Gansu Province Clinical Research Center for Digestive Diseases, Lanzhou, China

**Keywords:** Obesity, Abdominal obesity, Metabolic obesity phenotypes, *Helicobacter pylori* infection, NHANES

## Abstract

**Background:**

Recent studies on the association between *Helicobacter pylori* (*H. pylori*) infection and obesity have reported conflicting results. Therefore, the purpose of our study was to investigate the association of obesity, abdominal obesity, and metabolic obesity phenotypes with *H. pylori* infection.

**Methods:**

A cross-sectional study of 1568 participants aged 20 to 85 was conducted using the National Health and Nutrition Examination Survey (NHANES) cycle 1999–2000. Logistic regression models were employed to evaluate the association of general obesity as defined by body mass index (BMI), abdominal obesity as defined by waist circumference (WC) and waist-height ratio (WHtR), and metabolic obesity phenotypes with *H. pylori* seropositivity. Subgroup analyses stratified by age were conducted to explore age-specific differences in this association.

**Results:**

After grouping individuals according to their WHtR, the prevalence rate of WHtR ≥ 0.5 in *H. pylori-*seropositive participants was significantly higher than that in *H. pylori*-seronegative participants (79.75 vs. 68.39, *P* < 0.001). The prevalence of *H. pylori seropositivity* in non-abdominal obesity and abdominal obesity defined by WHtR was 24.97% and 31.80%, respectively (*P* < 0.001). In the subgroup analysis, the adjusted association between abdominal obesity, as defined by the WHtR, and *H. pylori* seropositivity was significant in subjects aged < 50 years (OR = 2.23; 95% CI, 1.24–4.01; *P* = 0.01) but not in subjects aged ≥ 50 years (OR = 0.84; 95% CI, 0.35–1.99; *P* = 0.66). Subjects older than 50 years old had an OR (95% CI) for metabolically healthy obesity of 0.04 (0.01–0.35) compared with the control group. *H. pylori* seropositivity was consistently not associated with obesity as defined by BMI.

**Conclusions:**

Abdominal obesity, as defined by the WHtR, was associated with *H. pylori* infection in subjects aged ≤ 50 years.

## Introduction

*Helicobacter pylori* (*H. pylori*) is a flagellated gram-negative bacterium [1, 2]. From January 2000 to June 2017, data from 73 countries showed that the prevalence of *H. pylori* infection was 44.3%, which was higher in developing countries than in developed countries (50.8% vs. 34.7%) [3]. It is well known that gastrointestinal diseases such as chronic gastritis, gastric cancer, and mucosa-associated lymphoid tissue lymphoma are linked to *H. pylori* [4]. According to current research, *H. pylori* infection is closely linked to various diseases outside the digestive tract [5], including diabetes mellitus (DM) and nonalcoholic fatty liver disease (NAFLD).

Obesity refers to the excessive accumulation of body fat. Obesity, a chronic metabolic disease, is the main component of the metabolic syndrome. Diseases caused by obesity include T2DM, hypertension, obstructive sleep apnea, and myocardial infarction. Over the past 50 years, the prevalence of obesity has increased globally and reached epidemic levels [6]. A meta-analysis found that the overall prevalence of global central obesity increased, whereas the prevalence of obesity in young and male subjects increased significantly according to temporal trends [7]. Therefore, as an important public health problem, obesity has been given attention in many countries [8]. Treatment of these diseases will increase the additional load on the healthcare system [9]. Current studies on the association between *H. pylori* infection and obesity have shown conflicting results. Some studies showed a positive correlation [10–14], while others found no correlation or even a negative correlation [15–17].

The body mass index (BMI) was commonly employed as a measure of obesity in previous articles. However, there are some limitations in the assessment of obesity by BMI. BMI does not provide any data on body composition. It is particularly insufficient for estimating body mass in physically active persons and in athletes who are often overweight, with a higher proportion of lean body mass but without any excess fat. Many epidemiological studies investigated the predictive value of BMI for cardiometabolic risk factors and cardiovascular events and consistently showed that BMI had a lower discriminatory power than waist circumference (WC) and waist-height ratio (WHtR) to distinguish individuals with high muscle mass from those with excess fat or abdominal obesity [18, 19]. Some scholars believe that the WHtR is a better parameter for cardiovascular risk, DM, and obesity [20–23]. An analysis of baseline data from a Chinese prospective cohort suggested WHtR to be the best indicator for dyslipidemia and hyperglycemia when compared with BMI and WC [24]. In recent years, a subtype of obesity that meets the diagnostic criteria for obesity without causing metabolic abnormalities such as T2DM or hyperlipidemia has been discovered. This obesity subtype is called metabolically healthy obesity (MHO). MHO is likely to progress to metabolically unhealthy obesity (MUO). In addition, there are metabolically healthy non-obese (MHN) and metabolically unhealthy non-obese (MUN) [25]. But there are few studies on the relationship between *H. pylori* infection and metabolic obesity phenotypes. Therefore, we assessed the cross-sectional association of general obesity, abdominal obesity, and metabolic obesity phenotypes with *H. pylori* seropositivity, utilizing data from the National Health and Nutrition Examination Survey (NHANES) conducted in the United States during 1999–2000.

## Methods

### Population

These data were obtained from the National Health and Nutrition Examination Survey (NHANES) cycle 1999–2000 because the participants in this cycle included data on *H. pylori* infection. The NHANES is a series of surveys led by the Center for Disease Control aimed at assessing the health and nutritional status of American adults and children since the 1960s. This was a complex, multi-stage probability sampling design using a nationally representative, non-institutionalized sample from the United States civilian population survey. The NHANES protocol was approved by the Institutional Review Board for Human Subjects of the United States Center for Disease Control and Prevention, and all participants provided written informed consent [26]. The data used for analysis in this study is publicly available on the NHANES website: https://www.cdc.gov/nchs/nhanes/index.htm.

**Fig. 1:**
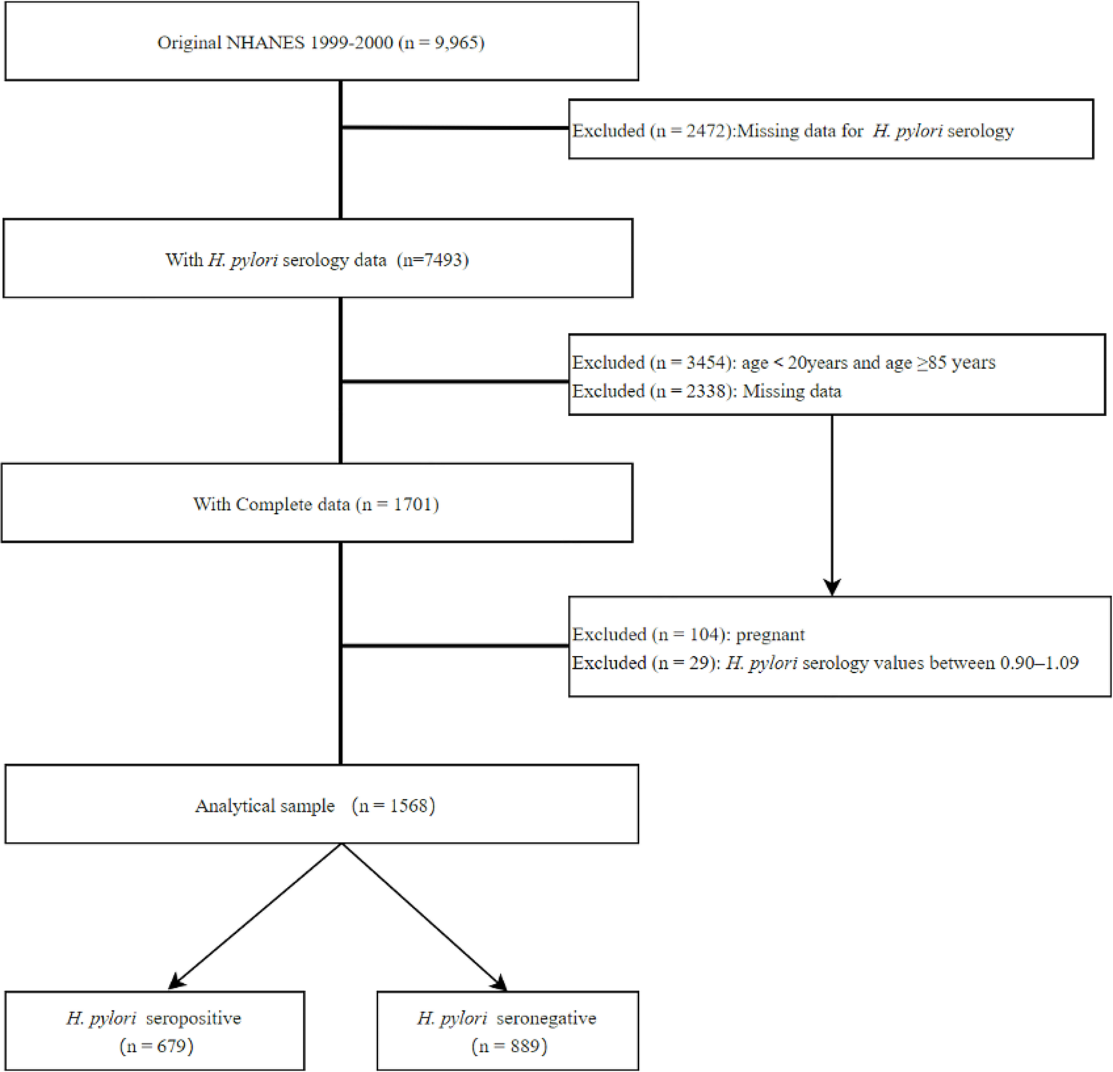
The sample selection flow chart

As is shown in Fig. 1, a total of 9965 subjects were selected. After excluding individuals who were pregnant and those without anthropometric measurements or serum biochemical parameters. Our study sample comprised 1568 U.S. citizens.

### Helicobacter pylori seropositivity

*H. pylori*-specific immunoglobulin G (IgG) was determined using the Wampole Laboratories *H. pylori* IgG enzyme-linked immunosorbent assay (ELISA), which was designed to detect and qualitatively determine *H. pylori* IgG antibodies in human serum. Participants were divided into *H. pylori* seropositive (optical density (OD) ≥ 1.1) and seronegative (OD < 0.9) groups, using the standard ELISA cut-off value. To avoid misleading statistical results, ambiguous values (0.9–1.1) were excluded from the analysis [27, 28].

### Obesity and metabolic phenotypes

The exposure variables were BMI, WC, and WHtR. Anthropometric measurements were performed using standardized procedures. In brief, a stadiometer was used for height measurement and an electronic digital scale to measure body weight. WC was measured at the high point of the iliac crest at minimal respiration. BMI was calculated by dividing body weight (kg) by the square of height (kg/m^2^). BMI was calculated to the nearest 0.1 kg/m^2^ and categorized according to the recommended cut points as follows: normal and underweight (< 25.0 kg/m^2^), overweight (25.0–30.0 kg/m^2^), and obesity (≥ 30 kg/m^2^) [29]. Abdominal obesity was defined as WC and WHtR [30, 31]. Abdominal obesity was defined by WC according to the thresholds for the US population included in the 2005 revision of the National Cholesterol Education Program’s (NCEP) Adult Treatment Panel III (ATP III): WC < 102 cm was considered normal for men, and WC ≥ 102 cm was considered abdominal obesity. WC < 88 cm was considered normal for women, and WC ≥ 88 cm was considered abdominal obesity [31]. WHtR was defined as the ratio of waist (cm) and height (cm), and based on previous studies, a widely used cut-off point was used to classify normal (WHtR < 0.5) or abdominal obesity (WHtR ≥ 0.5) [21, 22, 32, 33]. The complete measurement techniques for these variables were easily accessed at www.cdc.gov/nchs/nhanes/.

Metabolically healthy or unhealthy status was defined in accordance with the criteria outlined in the NCEP/ATP III as the following risk factors [34]: (1) systolic blood pressure ≥ 130 mm Hg and/or a diastolic blood pressure ≥ 85 mm Hg, or prescription medication for hypertension; (2) triglyceride ≥ 150 mg/dL (1.7 mmol/L); (3) fasting plasma glucose ≥ 100 mg/dL (5.6 mmol/L) or anti-diabetic treatment; and (4) high-density lipoprotein (HDL) cholesterol < 40 mg/dL (1.0 mmol/L) in men and < 50 mg/dL (1.3 mmol/L) in women. We constituted four groups of patients according to metabolic obesity phenotypes: the MHN phenotype was defined as the absence of all metabolic risk factors and a BMI < 30 kg/m^2^; the MHO phenotype was defined as the absence of all metabolic risk factors and a BMI ≥ 30 kg/m^2^; the MUN phenotype was defined as the presence of one or more metabolic risk factors and a BMI < 30 kg/m^2^; and the MUO phenotype was defined as the presence of one or more metabolic risk factors and a BMI ≥ 30 kg/m^2^ [35].

### Variables

We collected data on age, gender, household size, race, educational level, height, waist circumference, weight, body mass index, waist-height ratio, smoking behavior, triglyceride, total cholesterol, high-density lipoprotein, fasting glucose, glycated hemoglobin A1c (HbA1c), and C-reactive protein. For covariates, age, gender, race, educational level, household size, WHtR, DM, hypertension, and smoking behavior were used as categorical variables. BMI, WC, systolic blood pressure, diastolic blood pressure, triglyceride, total cholesterol, high-density lipoprotein, fasting glucose, HbA1c, homeostasis model assessment of insulin resistance (HOMA-IR) value, and serum C reactive protein were used as continuous variables [27, 28, 36]. HOMA-IR was calculated using the following formula: fasting insulin [µU/mL] × fasting glucose [mmol/L] / 22.5.

All blood pressure data (systolic blood pressure and diastolic blood pressure) were measured by professionals certified for blood pressure measurements in the Mobile Examination Center (MEC). The blood pressure measurements were taken after the participants remained at rest for 5 minutes and measured three times in a row. Participants were considered to have hypertension for any of the following reasons: if they responded “yes” to the question “Have you ever been told by a doctor or other health professional that you have hypertension, also called high blood pressure?”; if they self-reported antihypertensive drug use; or if they had a high biological measurement value (systolic blood pressure ≥ 140 mm Hg and/or diastolic blood pressure ≥ 90 mm Hg). The participant was considered DM if they met at least one of the following criteria: (1) self-reported doctor-diagnosed DM; (2) plasma HbA1c level ≥ 6.5%; (3) fasting plasma glucose level ≥ 126 mg/dL [37]. A previous diagnosis of DM was obtained from self-reported medical conditions [38].

The participants were categorized as nonsmokers, former smokers, or current smokers on the basis of their responses to those questions. Nonsmokers were defined as those who reported at baseline that they had smoked < 100 cigarettes in their lives. Former smokers were defined as those who had previously smoked > 100 cigarettes in their lifetime but were not currently smoking. Current smokers were defined as those who had previously smoked > 100 cigarettes in their lifetime and were currently smoking.

### Statistical analysis

To compare the baseline characteristics of the study sample, we assessed continuous variables as mean ± standard deviation (SD) and categorical variables as numbers or percentages (n, %). We evaluated the differences between the two groups using the Student’s t-test and the chi-square test. After adjusting for covariates, logistic regression analysis was used to examine the association between anthropometric indices and *H. pylori* seropositivity in the crude analysis and ORs (95% CIs). All statistical analyses were performed using appropriate NHANES sampling weights.

## Results

### Characteristics of included subjects

Our study sample comprised 1568 subjects, of whom 889 were *H. pylori* seronegative and 679 were *H. pylori* seropositive. The overall prevalence of *H. pylori* infection was 43.30% (679/1568). The subjects with higher rates of *H. pylori* seropositivity included those who were younger than 50 years old, had lower levels of education, had four or more household members living with them, had lower levels of high-density lipoprotein, had higher levels of fasting glucose, WHtR, and HbA1c, and were current smokers (all *P* < 0.05). In the baseline survey, we did not find any significant difference in BMI or WC between the two groups (Table 1).

**Table 1:** Baseline characteristics of the study subjects

Characteristic	H.pylori seronegative	H.pylori seropositive	*P* value
	(*n* = 889)	(*n* = 679)	
Age (n, %)			**< 0.001**
<50	501 (68.98)	289 (55.84)	
≥ 50	388 (31.02)	390 (44.16)	
Gender (n, %)			0.57
Women	454 (50.73)	330 (48.98)	
Men	435 (49.27)	349 (51.02)	
Household size (n, %)			**0.03**
Small (1-3members)	627 (70.57)	416 (63.04)	
Large(4 or more members)	262 (29.43)	263 (36.96)	
Race (n, %)			**< 0.001**
Non-Hispanic black	130 (6.86)	142 (16.29)	
Non-Hispanic white	554 (81.98)	156 (47.56)	
Mexican American	147 (3.50)	295 (13.06)	
Other	58 (7.66)	86 (23.09)	
Educational level (n, %)			**< 0.001**
< 9th grade	74 (2.96)	217 (17.48)	
9-11th grade	130 (12.20)	174 (24.73)	
High school/GED	234 (28.81)	105 (21.43)	
Some college	245 (28.45)	125 (23.98)	
College or above	206 (27.58)	58 (12.38)	
Smoking behavior (n, %)			**0.02**
Nonsmokers	454 (49.98)	334 (45.49)	
Former smokers	258 (28.55)	184 (24.42)	
Current smokers	177 (21.47)	161 (30.10)	
BMI (kg/m^2^)	27.60 (0.38)	27.73 (0.31)	0.73
WC (cm)	94.73 (0.93)	94.38 (0.82)	0.70
WHtR (n, %)			**< 0.001**
<0.5	223 (31.61)	97 (20.25)	
≥0.5	666 (68.39)	582 (79.75)	
TG (mmol/L)	1.56 (0.06)	1.65 (0.06)	0.25
TC (mmol/L)	5.24 (0.06)	5.24 (0.08)	0.96
HDL (mmol/L)	1.30 (0.02)	1.24 (0.03)	**0.04**
Fasting glucose (mmol/L)	5.46 (0.06)	5.82 (0.12)	**0.01**
HbA1c (%)	5.29 (0.06)	5.56 (0.07)	**0.002**
HOMA-IR value	3.10 (0.18)	3.30 (0.19)	0.35
CRP (mg/dL)	0.41 (0.03)	0.43 (0.03)	0.72
Systolic BP (mm Hg)	120.08 (0.74)	125.12 (1.44)	**0.01**
Diastolic BP (mm Hg)	73.32 (0.55)	72.61 (0.61)	0.45
Hypertension (n, %)			0.05
No	480 (61.48)	306 (53.26)	
Yes	409 (38.52)	373 (46.74)	
DM (n, %)			0.13
No	585 (71.42)	396 (65.71)	
Yes	304 (28.58)	283 (34.29)	
Metabolic obesity phenotypes (n, %)			0.09
MHN	181 (25.13)	90 (16.77)	
MHO	36 (3.24)	16 (2.85)	
MUN	420 (45.91)	369 (54.84)	
MUO	252 (25.72)	204 (25.54)	

BMI: body mass index; WC: waist circumference; WHtR: Waist-height ratio; TG: Triglycerides; TC: Total cholesterol; HDL: high-density lipoprotein; CRP: C reactive protein; BP: blood pressure; DM: diabetic mellitus;

Hypertension: Systolic BP ≥ 140 mm Hg or Diastolic BP ≥ 90 mm Hg

Bold represents statistically significant

### The prevalence of *H. Pylori* seropositivity after grouping according to BMI, WC and WHtR

**Fig. 2:**
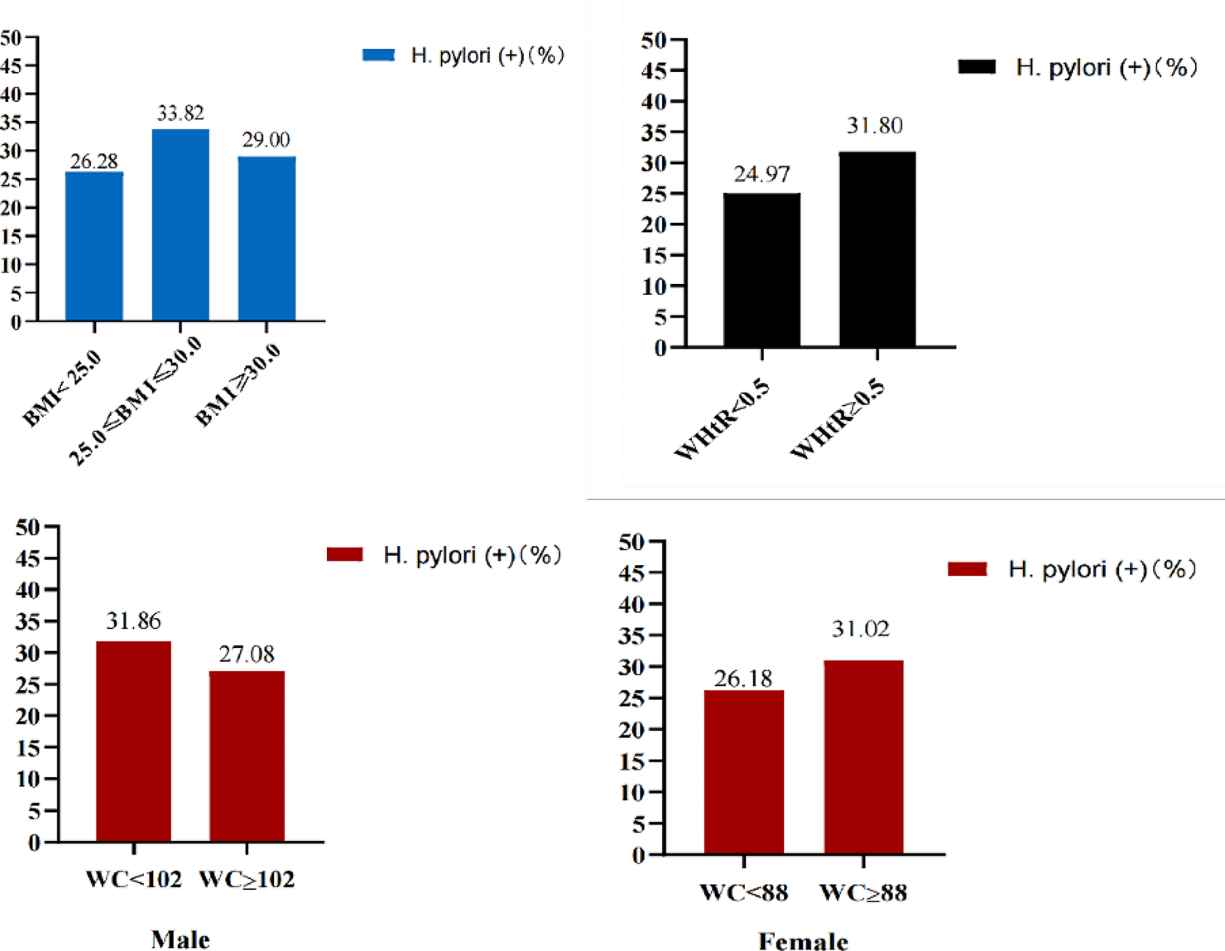
The prevalence of *H. pylori* seropositivity (*H. pylori* (+)) after grouping according to BMI, WC and WHtR

Figure 2 indicates the prevalence of *H. pylori seropositivity* after grouping individuals. The blue bars indicate that the prevalence of *H. pylori seropositivity* in the normal and underweight (< 25.0 kg/m^2^), overweight (25.0–30.0 kg/m^2^), and obese (≥ 30 kg/m^2^) subjects was 26.28%, 33.82%, and 29.00%, respectively (*p*  = 0.262). The red bars indicate that the prevalence of *H. pylori seropositivity* in non-abdominal obesity and abdominal obesity defined by WC was 31.86% and 27.08% in males (*p*  = 0.204) and 26.18% and 31.02% in females (*p*  = 0.259), respectively. The black bars indicate that the prevalence of *H. pylori seropositivity* in non-abdominal obesity and abdominal obesity defined by WHtR was 24.97% and 31.80%, respectively (*p*  < 0.001).

### *H. Pylori* seropositivity *and general obesity*

We used logistic regression analysis to assess the association between *H. pylori* seropositivity and BMI stratification, adjusted for confounding factors, and determined the odds ratios for being overweight or obese based on *H. pylori* infection status. Among all subjects, the OR (95% CI) for being overweight in Model 1 was 1.43 (0.99–2.07), compared with normal and underweight, but the statistical significance disappeared after adjustment for additional covariates in Models 2, 3, and 4. The OR (95% CI) for being overweight among subjects younger than 50 years was significant only in Model 1, at 1.67 (1.04–2.66); being overweight or obese was not associated with *H. pylori* seropositivity among subjects older than 50 years. In summary, no association was observed between being overweight or obese and *H. pylori* seropositivity after adjusting for covariates (Table 2).

**Table 2:** The association between body mass index and *H. pylori* seropositivity

All subjects	Model 1 OR (95% CI)	*P* value	Model 2 OR(95% CI)	*P* value	Model 3 OR(95% CI)	*P* value	Model 4 OR(95% CI)	*P* value
Normal and underweight	Ref		Ref		Ref		Ref	
Overweight	1.43 (0.99, 2.07)	0.05	0.98 (0.47, 2.01)	0.90	1.06 (0.71, 1.58)	0.75	1.26 (0.77, 2.06)	0.34
Obesity	1.15 (0.85, 1.55)	0.34	0.79 (0.47, 1.34)	0.20	0.88 (0.72, 1.08)	0.21	1.39 (0.76, 2.53)	0.26
Subjects aged < 50 years								
Normal and underweight	Ref		Ref		Ref		Ref	
Overweight	1.67 (1.04, 2.66)	0.04	1.17 (0.63, 2.19)	0.47	1.25 (0.86, 1.83)	0.23	1.46 (0.86, 2.47)	0.15
Obesity	1.39 (0.93, 2.10)	0.10	0.98 (0.62, 1.55)	0.92	1.02 (0.74, 1.42)	0.87	1.64 (0.67, 4.00)	0.25
Subjects aged ≥ 50 years								
Normal and underweight	Ref		Ref		Ref		Ref	
Overweight	0.98 (0.62, 1.55)	0.93	0.77 (0.35, 1.71)	0.38	0.80 (0.42, 1.54)	0.48	0.90 (0.44, 1.85)	0.75
Obesity	0.72 (0.47, 1.12)	0.13	0.60 (0.30, 1.22)	0.11	0.63 (0.39, 1.02)	0.06	0.81 (0.39, 1.72)	0.56

Model 1: no covariates were adjusted;

Model 2: gender, age, race, educational level, and household size were adjusted;

Model 3: adjusted for variables in model 2, plus smoking behavior, DM, and blood pressure were adjusted;

Model 4: adjusted for variables in model 3, plus WC, triglycerides, total cholesterol, high-density lipoprotein, fasting glucose, HbA1c, HOMA-IR, and serum C reactive protein

### *H. Pylori* seropositivity *and abdominal obesity*

Among all subjects, the OR (95% CI) for subjects with a WHtR ≥ 0.5 in Model 1 was 1.82 (1.37–2.41), compared with a WHtR of < 0.5, but the statistical significance disappeared after adjustment for additional covariates in Models 2, 3, and 4. Abdominal obesity defined by the WHtR was not associated with *H. pylori* seropositivity among subjects older than 50 years; these findings did not change after adjusting for covariates in models 2, 3, and 4 (Table 3). Among subjects aged ≤ 50 years, the OR (95% CI) for subjects with WHtR ≥ 0.5 in Model 1 was 1.87 (1.35–2.58) compared with the control group. This finding was still statistically significant in Models 2, 3, and 4, with ORs (95% CI) of 1.47 (0.99–2.18), 1.67 (1.16–2.41), and 2.23 (1.24–4.01), respectively. However, we did not observe an association between *H. pylori* seropositivity and abdominal obesity as defined by WC (Table 4).

**Table 3:** The association between waist-to-height ratio and *H. pylori* seropositivity

All subjects	Model 1 OR (95% CI)	*P* value	Model 2 OR (95% CI)	*P* value	Model 3 OR (95% CI)	*P* value	Model 4 OR (95% CI)	*P* value
< 0.5	Ref		Ref		Ref		Ref	
≥ 0.5	1.82 (1.37, 2.41)	< 0.001	1.17 (0.77, 1.78)	0.33	1.31 (0.96, 1.81)	0.09	1.58 (0.98, 2.55)	0.06
Subjects aged < 50 years								
< 0.5	Ref		Ref		Ref		Ref	
≥ 0.5	1.87 (1.35, 2.58)	**0.001**	1.47 (0.99, 2.18)	**0.05**	1.67 (1.16, 2.41)	**0.01**	2.23 (1.24, 4.01)	**0.01**
Subjects aged ≥ 50 years								
< 0.5	Ref		Ref		Ref		Ref	
≥ 0.5	1.01 (0.59, 1.72)	0.98	0.78 (0.33, 1.83)	0.46	0.80 (0.40, 1.58)	0.49	0.84 (0.35, 1.99)	0.66

Model 1: no covariates were adjusted;

Model 2: gender, age, race, educational level, and household size were adjusted;

Model 3: adjusted for variables in model 2, plus smoking behavior, DM, and blood pressure were adjusted;

Model 4: adjusted for variables in model 3, plus BMI, triglycerides, total cholesterol, high-density lipoprotein, fasting glucose, HbA1c, HOMA-IR, and serum C reactive protein

**Table 4:** The association between waist circumference and *H. pylori* seropositivity

All subjects	Model 1 OR (95% CI)	*P* value	Model 2 OR (95% CI)	*P* value	Model 3 OR (95% CI)	*P* value	Model 4 OR (95% CI)	*P* value
Subjects aged < 50 years								
Male								
< 102	Ref		Ref		Ref		Ref	
≥ 102	0.63 (0.38, 1.03)	0.06	0.46(0.17,1.29)	0.11	0.46(0.18,1.18)	0.10	0.27(0.07,1.02)	0.05
Female								
< 88	Ref		Ref		Ref		Ref	
≥ 88	0.72 (0.43, 1.22)	0.20	0.46 (0.26, 0.81)	0.02	0.44 (0.23, 0.82)	0.01	0.49 (0.19, 1.26)	0.13
Subjects aged ≥ 50 years								
Male								
< 102	Ref		Ref		Ref		Ref	
≥ 102	0.87 (0.50, 1.52)	0.59	0.89 (0.42, 1.85)	0.69	0.90 (0.45, 1.79)	0.74	0.73 (0.32, 1.67)	0.43
Female								
< 88	Ref		Ref		Ref		Ref	
≥ 88	1.38 (0.78, 2.46)	0.25	1.09 (0.54, 2.19)	0.76	1.01 (0.60, 1.71)	0.97	0.96 (0.38, 2.41)	0.93

Model 1: no covariates were adjusted;

Model 2: race, educational level, and household size were adjusted;

Model 3: adjusted for variables in model 2, plus smoking behavior, DM, blood pressure were adjusted;

Model 4: adjusted for variables in model 3, plus BMI, triglycerides, total cholesterol, high-density lipoprotein, fasting glucose, HbA1c, HOMA-IR, and serum C reactive protein

### *H. Pylori* seropositivity *and* metabolic obesity phenotypes

We analyzed the data to investigate the relationships between metabolic obesity phenotypes and *H. pylori* seropositivity. The OR (95% CI) for MUN was significant only in Model 1 among all subjects, at 1.79 (1.12–2.86). In Model 1, subjects older than 50 years had an OR (95% CI) of 0.09 (0.01–0.69) of the MHO phenotype, compared with the control group; after adjustment for major covariates, their ORs (95% CI) were 0.05 (0.00–2.87), 0.04 (0.01–0.35), and 0.04 (0.01–0.35) in Models 2, 3, and 4, respectively (Table 5). However, among subjects younger than 50 years, none of the models revealed any statistical significance related to *H. pylori* seropositivity.

**Table 5:** The association between metabolic obesity phenotypes and *H. pylori* seropositivity

All subjects	Model 1 OR (95% CI)	*P* value	Model 2 OR (95% CI)	*P* value	Model 3 OR (95% CI)	*P* value	Model 4 OR (95% CI)	*P* value
MHN	Ref		Ref		Ref		Ref	
MUN	1.79 (1.12, 2.86)	0.02	1.20 (0.71, 2.04)	0.59	1.16 (0.69, 1.97)	0.54	1.20 (0.70, 2.06)	0.49
MHO	1.32 (0.49, 3.58)	0.56	0.59 (0.18, 1.90)	0.51	0.59 (0.18, 1.98)	0.37	0.67 (0.20, 2.22)	0.48
MUO	1.49 (0.90, 2.47)	0.11	0.97 (0.59, 1.61)	0.92	0.99 (0.61, 1.59)	0.95	1.09 (0.67, 1.78)	0.70
Subjects aged < 50 years								
MHN	Ref		Ref		Ref		Ref	
MUN	1.63 (0.99, 2.70)	0.05	1.38 (0.47, 4.10)	0.33	1.37 (0.81, 2.32)	0.22	1.49 (0.92, 2.40)	0.10
MHO	2.03 (0.65, 6.38)	0.20	0.93 (0.06, 13.48)	0.91	0.92 (0.23, 3.67)	0.90	1.09 (0.30, 3.91)	0.89
MUO	1.44 (0.83, 2.48)	0.17	1.16 (0.43, 3.13)	0.59	1.18 (0.74, 1.86)	0.46	1.57 (0.94, 2.62)	0.08
Subjects aged ≥ 50 years								
MHN	Ref		Ref		Ref		Ref	
MUN	1.16 (0.49, 2.77)	0.71	0.84 (0.12, 5.73)	0.73	0.83 (0.32, 2.13)	0.68	0.78 (0.29, 2.09)	0.59
MHO	0.09 (0.01, 0.69)	**0.02**	0.05 (0.00, 2.87)	**0.08**	0.04 (0.01, 0.35)	**0.01**	0.04 (0.01, 0.35)	**0.01**
MUO	0.90 (0.38, 2.12)	0.79	0.66 (0.09, 4.65)	0.45	0.67 (0.25, 1.82)	0.40	0.59 (0.20, 1.79)	0.33

MHN: metabolically healthy non-obese; MUN: metabolically unhealthy non-obese; MHO: metabolically healthy obese; MUO: metabolically unhealthy obese

Model 1: no covariates were adjusted;

Model 2: gender, age, race, educational level, and household size were adjusted;

Model 3: adjusted for variables in model 2, plus smoking behavior were adjusted

Model 4: adjusted for variables in model 3, plus total cholesterol, HOMA-IR, HbA1c, and serum C reactive protein

## Discussion

The purpose of our study was to investigate the association of obesity, abdominal obesity, and metabolic obesity phenotypes with *H. pylori* infection using the NHANES cycle 1999–2000. This study is the first to use NHANES data to prove that abdominal obesity, as defined by WHtR, is related to *H. pylori* seropositivity in subjects younger than 50 years old after adjusting for certain covariates compared to the control group. However, we did not find an association between *H. pylori* seropositivity and obesity in MUO individuals and overweight/obese individuals defined by BMI. We will explain these results later. Recently, studies on the relationship between *H. pylori* infection and obesity have been conducted. A large-scale cross-sectional study involving 76,915 participants in West China found that the prevalence of *H. pylori* infection in subjects with abdominal obesity (42.20%) was significantly higher than that in subjects with normal WC (39.10%) [10]. A cohort study in Israel involving 235,107 individuals showed that *H. pylori* positivity was positively associated with an increased BMI, or overweight/obesity [11]. Chen et al. conducted a study on 2604 subjects in Taiwan and discovered that subjects with *H. pylori* infection and those aged less than 50 years may have an increased risk of obesity (BMI ≥ 30) compared to those without this type of infection [12]. Similarly, a cross-sectional study showed that the prevalence of *H. pylori* infection in obese individuals was higher than that in individuals with a low BMI (BMI < 25 kg/m2) [13]. A recent meta-analysis showed that the risk of *H. pylori* infection was positively correlated with the prevalence of obesity [14]. According to the existing evidence, the relationship between *H. pylori* infection and obesity may involve several mechanisms. First, *H. pylori* may affect gut hormones involved in food intake and energy expenditure, such as ghrelin, obestatin, and leptin. Some studies have reported that serum leptin levels are significantly reduced in patients with *H. pylori* infection [39, 40]. Low levels of leptin delay satiety while eating, causing excessive nutrient intake and obesity [41]. Second, insulin resistance is a significant risk factor for metabolic disorders. *H. pylori* may contribute to metabolic disorders and obesity by producing pro-inflammatory cytokines, such as tumor necrosis factor-α, interleukin (IL)-1, IL-6, and IL-8, which increase inflammatory responses and promote insulin resistance [42, 43]. The third factor is that the intestinal immune function of obese people is impaired, and the ability of monocytes to transform into macrophages is reduced in these patients. Meanwhile, the cytotoxic activity of natural killer cells in obese individuals is lower than that in healthy individuals with a BMI [44]. These two points may indicate that the immune environment of obese individuals promotes *H. pylori* survival [45, 46].

Abdominal obesity, as defined by WHtR, was associated with *H. pylori* infection in subjects younger than 50 years old compared with the control group in our study. As mentioned above, a meta-analysis has shown that the prevalence of central obesity among young subjects has increased significantly [7]. Our study found that the correlation between *H. pylori* seropositivity and obesity was most significant among young subjects. Therefore, we believe that this may be related to the fact that most subjects were infected with *H. pylori* when they were young. The inflammatory responses generated by *H. pylori* may be more active during this period and may have a greater impact on insulin resistance [12]. In older subjects, more diseases lead to systemic inflammatory responses, which can attenuate the effects of *H. pylori* infection on obesity [47–49]. In addition, we explored the relationship between metabolic obesity phenotypes and *H. pylori*, and the results showed that MHO ≥ 50 years subjects seem protected against *H. pylori* infection seropositivity (OR(95% CI): 0.04 (0.01, 0.35), *P* = 0.01). It suggests that the comorbidities present in obesity could be related to the *H. pylori* infection in middle-aged and elderly subjects. However, some studies did not find any clinical correlation between *H. pylori* infection and obesity. A study aimed at exploring the association between *H. pylori* infection and obesity or weight gain in a Chinese population did not observe a correlation between *H. pylori* infection and obesity after adjusting for confounders (RR: 0.831, 95% CI: 0.577–1.197, *P* = 0.321) [15]. A cross-sectional analysis of two separate populations of older adults (*n* = 13,044) from the Netherlands and Germany was conducted. Meta-analysis of cross-sectional data revealed no association between anti-*H. pylori* IgG titer and BMI, nor between *H. pylori* positivity and BMI. Mendelian randomization showed no causal relation between *H. pylori* genetic risk score and BMI or obesity, nor between BMI or obesity genetic risk scores and *H. pylori* positivity [16]. Interestingly, a retrospective study compared the prevalence of *H. pylori* colonization between obese and healthy-weight children. The results showed that *H. pylori* colonization was 10% in obese children compared with 21% in healthy-weight children (RR = 2.1, 95% CI, 1.1–4.0). Multivariate analysis showed that *H. pylori* colonization was associated with a 50% reduction in the odds of obesity (adjusted OR = 0.5, 95% CI, 0.2–1.0) [17]. Even some studies have found that eradicating *H. pylori* infection increases the incidence of obesity [50–53]. In the above studies, different methods were used to detect *H. pylori*. Therefore, we believe that these inconsistent findings may be related to the following reasons: firstly, different detection methods for *H. pylori* infection will affect the research results. Because of its advantages of being non-invasive, rapid, and low-cost, the stool antigen test (SAT) is recommended for the preliminary diagnosis of *H. pylori* infection; the sensitivity and specificity of the test were 95.5% and 97.6%, respectively [54]. A study found that the sensitivity and specificity of the urea breath test (UBT) were 96% and 93%, respectively, which was similar to the accuracy of the SAT using ELISA [55]. The *H. pylori* antibody test in urine only detects exposure status, with a specificity of 91% [56]. In our study, serological testing was used to diagnose *H. pylori* infection because it is non-invasive and fast. However, the diagnostic accuracy of serological tests is poor. One study demonstrated sensitivity ranging from 76 to 84% and specificities from 79 to 90% [57]. Different detection methods have different sensitivity and specificity, which causes the difference in the diagnosis rate of *H. pylori*, thus affecting the result. In addition, it cannot distinguish an active infection from a previous infection because antibodies continue to exist in the blood after eradication [58]. Second, infections with different *H. pylori* strains may have produced inconsistent results. For example, one study found that the prevalence of *H. pylori* infection and CagA strains differed among Americans of various races [59]. Lastly, this study used different measures to define obesity, and the findings suggest that *H. pylori* is associated with obesity as defined by WHtR but not with general obesity as defined by BMI or abdominal obesity as defined by WC, which may be related to the following reasons: WHtR is a more accurate body fact anthropometric index to define obesity. A study of 4052 participants was conducted to investigate the association between four anthropometric measures and the prevalence of diabetes. The research shows that the WHtR was more closely related to diabetes than BMI and WHR among study participants [60]. A meta-analysis showed that WHtR was a better predictor than WC and BMI for diabetes, dyslipidemia, hypertension, and cardiovascular disease risk in both sexes in populations of various nationalities and ethnic groups, and WHR should be considered as a screening tool [23]. This also led to a positive correlation between abdominal obesity defined by WHR and *H. pylori* infection seropositivity in subjects less than 50 years old in this study.

The strength of our study is that our data came from a nationally representative sample, and we also used different anthropological measures, so our results can provide strong evidence to determine the relationship between obesity and *H. pylori* infection. There are some limitations to our study. First, because this was a cross-sectional study, we could not ascertain the causal relationship between obesity and *H. pylori* infection. Second, serological testing cannot indicate that the patient is currently infected, which may reduce the rate of *H. pylori* infection. Finally, the relationship between the different *H. pylori* strains and obesity could not be explored.

In conclusion, we report that abdominal obesity, as defined by the WHtR, is associated with *H. pylori* infection in subjects aged ≤ 50 years. Whereas MUO and overweight/obese individuals (defined by BMI) were not associated with *H. pylori* infection.

## Data Availability

The datasets analyzed in this study are available in the NHANES repository (https://wwwn.cdc.gov/nchs/nhanes/Default.aspx).
